# The Water Treatment of Burns

**Published:** 1909-08-28

**Authors:** 


### Surgery.

### THE WATER TREATMENT OF BURNS.

The water treatment—or " hydriatic management "—of burns is a modification of an old-fashioned and useful procedure known as the wet-bandage method. According to authorities such as Dzondi, Winternitz, Grosse, and others who have used it extensively, it is superior to all other methods in stilling pain, saving lives, and securing good results with a minimum of deformity from scarring. It is easily performed, it is cheap, and it can be undertaken everywhere. Grosse puts it thus: " I heartily join in the hope of Dzondi and Winternitz, that hydriatic management of burns will be the treatment of the future, for the sake of suffering humanity." He points out that in regard to the principles of the treatment of wounds it is generally recognised that that procedure is best which prevents infection, most thoroughly removes secretion, and reduces irritation as far as possible. Finally, the treatment should favour the delicate microscopic processes that occur in the wound and lead to recovery.

Powders and ointments do not fulfil these demands. The former dry up and then form a shield under which germs flourish. Ointments check the removal of secretions more than they favour it. The undesired by-effects of powders and salves have induced many surgeons to prefer wet bandages. The question is, how is a wet compress to be administered to comply with the desiderata given above? It too often happens that the bactericidal power of the applications absorbs attention so that the irritation and other ill-effects exerted upon the wound are overlooked. One should always bear in mind the fact that an agent when killing microbes or preventing them from developing will act likewise upon the growing and proliferating cells of the tissues. Frequent changing of the dressings may be advocated, but surgeons are too apt to employ uselessly concentrated lotions, whereby the vitality of the superficial cell-layers becomes greatly lowered. There is no objection to applying a somewhat higher concentration at first, but very soon better results are obtained by saturating the frequently changed compresses with more dilute solutions. Whatever harm is liable to accrue from the low concentration is overbalanced by the fact that the frequent changing of the compresses brings new quantities of the disinfectant into direct contact with the infected parts. Dr. Grosse advocates no stronger lotions than chinosol, 1 in 2,000; alum acetate, 0.4 per cent.; protargol, 0.25 per cent.; boric acid, $\frac{1}{3}$ to $\frac{1}{2}$ of the concentrated solution; or, for abundant granulations, zinc chloride 1 in 600.

If so wide a surface of skin is burned that a partial bath is impossible, the patient is placed in a full bath, the temperature of which should be from 80° F. to 88° F. to 93° F. The larger the affected area the warmer as a rule should the water be; it may be regulated a good deal according to the patient's feeling and the requirements of the case. After the accident the clothing should not be removed, except for the boots or such loose articles as can be taken off without a great deal of disturbance. As soon as the first shock and the worst pain are over and all preparations for the continuous bath have been made, the patient should be undressed.

If the burn be local, affecting some portion of the body that admits of a partial bath, only this part is placed in a suitable vessel, again without undressing and without cleansing, except, of course, when the burn has been due to caustic chemicals. The temperature of the local bath may be from 70° F. to 85° F. Extreme degrees should be avoided; the feelings of the patient afford much help in deciding the best temperature in the case. The duration may be from fifteen minutes up to days, the latter in necrosing cases. When the worst of the pain has been relieved the clothes may be removed and the part cleaned, during which process all disinfectants are to be avoided, and even the cleansing done in a somewhat perfunctory manner. Blisters should not be opened. The partial bath may then be continued at a higher temperature—90° F. to 99° F.—for a longer time, or the part dressed as detailed below. When no bath is available, if the whole body is more or less affected, a woollen blanket is spread upon a bed, with rubber tissue over it, and over this again two wet bed sheets, the patient being lifted up on to the latter. The upper sheet is now cut so that each part of the body can be covered separately by wrapping the strips around the parts, or by gently pressing the pieces smoothly upon the surface. One end of the bedstead is elevated a little, and water at 50° F. to 70° F. is poured from time to time without pressure over the aching parts. If the pain ceases, the underlying wet sheets and dry blankets may be closed over the patient to warm him. Cold extremities may be avoided by the use of hot bottles of indiarubber. In case this management is impossible in emergency, the water is simply poured upon the burned undressed patient, or if unclothed he is covered with some cloth to prevent all mechanical irritation. In burns of the first degree the treatment so far outlined will be sufficient. In widespread burns of the second and third degrees the patient is afterwards put into the continuous bath as already described.

If only a part of the body has been burned, this part should be undressed and, without disinfection and cleansing, covered tightly and smoothly with one or two wet layers of fine linen, muslin, or similar material, or two or three wet layers of plain absorbent gauze. According to the locality—for instance, around an extremity—a gauze roller may be wound twice, or these layers may be placed only upon the burned area where rollers cannot be used. These two primary layers remain upon the burn for from one to two days. The object is to save the part from mechanical irritation. Upon them are applied cold compresses wrung out of water at 50° F. to 70° F., removable whenever the pain increases. This means very often at first, even so often as every other minute; but subsequently the intervals lengthen. If a cooling coil is at hand it may be used with water at a higher temperature up to 65° F., or even 75° F. Extreme warmth over the burned area does harm, and so do ice bags, although, if the burn is upon the hand, forearm, or lower part of the leg, it may be useful to apply ice in one form or another to the healthy part near the burn in addition to the local dressing.

In burns of the first or second degree this treatment will suffice. Blisters, if not too large, will be absorbed within one or two days, or may be then opened and cleared away. The pain caused by the burn at first will by now be gone. In burns of the third degree, however, pus now begins to appear, and inflammatory pain with an inflammatory halo around the burned area. This is the time to remove the "primary layers," as they have been termed above. Pieces of plain absorbent gauze, five to ten layers, according to the quantity of oozing, are now cut of such shape and size as to cover the burned region exactly. The gauze is wrung out of one of the dilute antiseptic solutions already mentioned, placed upon the wound, and covered with a soft impervious stuff one inch or so larger than the gauze. The dressing is then fastened by a roller that presses the impervious cover all round against the healthy skin in order to prevent drying of the wet gauze. This dressing has to be removed whenever pain arises or the gauze is saturated with pus. Instead of impervious covering and gauze roller it may be preferable to apply over the wet medicated gauze a common wet compress—a towel wrung out of plain water—covered with a woollen cloth or flannel—not with an impervious material. This applies, for instance, to burned fingers where the medicated gauze is applied to each finger separately and the towel over the whole hand.

